# Daily Mean Temperature and Clinical Kidney Stone Presentation in Five U.S. Metropolitan Areas: A Time-Series Analysis

**DOI:** 10.1289/ehp.1307703

**Published:** 2014-07-10

**Authors:** Gregory E. Tasian, Jose E. Pulido, Antonio Gasparrini, Christopher S. Saigal, Benjamin P. Horton, J. Richard Landis, Rodger Madison, Ron Keren, for the Urologic Diseases in America Project

**Affiliations:** 1 Division of Urology, Department of Surgery, The Children’s Hospital of Philadelphia, Philadelphia, Pennsylvania, USA; 2 Division of Urology, Department of Surgery, Perelman School of Medicine at The University of Pennsylvania, Philadelphia, Pennsylvania, USA; 3 Center for Pediatric Clinical Effectiveness, The Children’s Hospital of Philadelphia, Philadelphia, Pennsylvania, USA; 4 Department of Medical Statistics, London School of Hygiene and Tropical Medicine, London, England; 5 Department of Urology, University of California, Los Angeles, Los Angeles, California; 6 RAND Corporation, Santa Monica, California, USA; 7 Department of Marine and Coastal Sciences, Rutgers University, New Brunswick, New Jersey, USA; 8 Division of Earth Sciences and Earth Observatory of Singapore, Nanyang Technological University, Singapore; 9 Department of Epidemiology and Biostatistics, Perelman School of Medicine at The University of Pennsylvania, Philadelphia, Pennsylvania, USA; 10 Department of Pediatrics, The Children’s Hospital of Philadelphia, Philadelphia, Pennsylvania, USA

## Abstract

Background: High ambient temperatures are a risk factor for nephrolithiasis, but the precise relationship between temperature and kidney stone presentation is unknown.

Objectives: Our objective was to estimate associations between mean daily temperature and kidney stone presentation according to lag time and temperatures.

Methods: Using a time-series design and distributed lag nonlinear models, we estimated the relative risk (RR) of kidney stone presentation associated with mean daily temperatures, including cumulative RR for a 20-day period, and RR for individual daily lags through 20 days. Our analysis used data from the MarketScan Commercial Claims database for 60,433 patients who sought medical evaluation or treatment of kidney stones from 2005–2011 in the U.S. cities of Atlanta, Georgia; Chicago, Illinois; Dallas, Texas; Los Angeles, California; and Philadelphia, Pennsylvania.

Results: Associations between mean daily temperature and kidney stone presentation were not monotonic, and there was variation in the exposure–response curve shapes and the strength of associations at different temperatures. However, in most cases RRs increased for temperatures above the reference value of 10°C. The cumulative RR for a daily mean temperature of 30°C versus 10°C was 1.38 in Atlanta (95% CI: 1.07, 1.79), 1.37 in Chicago (95% CI: 1.07, 1.76), 1.36 in Dallas (95% CI: 1.10, 1.69), 1.11 in Los Angeles (95% CI: 0.73, 1.68), and 1.47 in Philadelphia (95% CI: 1.00, 2.17). Kidney stone presentations also were positively associated with temperatures < 2°C in Atlanta, and < 10°C in Chicago and Philadelphia. In four cities, the strongest association between kidney stone presentation and a daily mean temperature of 30°C versus 10°C was estimated for lags of ≤ 3 days.

Conclusions: In general, kidney stone presentations increased with higher daily mean temperatures, with the strongest associations estimated for lags of only a few days. These findings further support an adverse effect of high temperatures on nephrolithiasis.

Citation: Tasian GE, Pulido JE, Gasparrini A, Saigal CS, Horton BP, Landis JR, Madison R, Keren R, for the Urologic Diseases in America Project. 2014. Daily mean temperature and clinical kidney stone presentation in five U.S. metropolitan areas: a time-series analysis. Environ Health Perspect 122:1081–1087; http://dx.doi.org/10.1289/ehp.1307703

## Introduction

Nephrolithiasis (kidney stones) is a painful condition that recurs in 50% of patients (Johnson et al. 1979) and is associated with kidney function loss, including end stage renal disease (Alexander et al. 2012). The prevalence of nephrolithiasis has increased markedly in Europe, Asia, and the Americas over the last three decades (Romero et al. 2010).

The etiology of kidney stones is multifactorial, but one important risk factor is high ambient temperature. Observed geographic and seasonal differences in nephrolithiasis rates (Curtin and Sampson 1989; Fakheri and Goldfarb 2009; Soucie et al. 1996) and urinary calcium and oxalate excretion (Elomaa et al. 1982) implicate high ambient temperature in the causal pathway (Fakheri and Goldfarb 2011). High ambient temperatures cause water loss, urinary concentration, and low urine volume and pH. This increases the relative supersaturation of calcium and uric acid, which thereby promotes nucleation, growth, and aggregation of lithogenic minerals in urine (Masterson et al. 2013; Parks et al. 2003). Consistent with this mechanism, previous studies suggested that high environmental temperatures are associated with short-term increases in the risk of nephrolithiasis (Boscolo-Berto et al. 2008; Evans and Costabile 2005; Fletcher et al. 2012). However, these studies were limited by the assessment of temperatures only in summer or extreme conditions (Evans and Costabile 2005; Fletcher et al. 2012), inclusion of subjects from similar geographic areas (Boscolo-Berto et al. 2008; Evans and Costabile 2005; Fletcher et al. 2012) or of selected patients admitted to the hospital (Fletcher et al. 2012), and assessment of lag periods between exposure and outcome that may have missed significant delayed associations between high daily temperatures and kidney stone presentation (Boscolo-Berto et al. 2008; Fletcher et al. 2012). The precise relationship between temperature and nephrolithiasis thus remains uncertain.

By 2100, global average temperatures are estimated to increase by 1–4.5°C due to increases in greenhouse gas emissions (Meinshausen et al. 2011; Solomon et al. 2007). With the continued threat of climate change, it is important to quantify the impact of temperature on nephrolithiasis. High daily temperatures increase the risk of cardiopulmonary death (Guo et al. 2011), cardiovascular death (Armstrong 2006; Smoyer et al. 2000), and acute renal failure (Fletcher et al. 2012). It is possible that a similar relationship exists for kidney stones.

Our objective was to define the overall cumulative exposure–response and the lag response relationships between daily temperatures and kidney stone presentation in five major American cities with diverse climates.

## Methods

*Data sources*. This study was conducted among an insured population using the MarketScan Commercial Claims database (Truven Health Analytics; http://truvenhealth.com/your-healthcare-focus/Life-Sciences/MarketScan-Databases-and-Online-Tools). MarketScan contains claims data from 2005 for 95 million unique patients enrolled in > 100 nongovernmental health insurance plans in all states. All data is deidentified and each enrollee is assigned a unique identifier. The databases contain demographic information such as age, sex, dates of services, *International Classification of Disease, Revision 9* (ICD-9) codes, and Current Procedural Terminology (CPT) codes (American Medical Association), but not race. Data of the enrollee’s geographic location are available at the 3-digit ZIP code and metropolitan statistical area (MSA) levels. The present study was exempt from institutional review board review per Department of Health and Human Services regulation 45 CFR 46.101, category 4.

Weather data were obtained from the National Weather Service United States Air Force–Navy weather stations (National Climatic Data Center; http://www.ncdc.noaa.gov/). We determined the mean, minimum, and maximum daily (24-hr) temperatures and the mean daily relative humidity by averaging the hourly recordings for all weather stations within each city. Stations with missing hourly data were excluded from that day’s values.

*Study population*. The eligible population comprised adults and children living in the MSAs of the U.S. cities of Atlanta (Georgia), Chicago (Illinois), Dallas (Texas), Los Angeles (California), and Philadelphia (Pennsylvania) between 2005 and 2011. These cities represent climate zones in which 30% of the world population lives (Mellinger et al. 1999). Atlanta, Dallas, and Philadelphia have humid subtropical climates with hot summers and mild-to-cool winters. Chicago has a continental climate with hot summers and cold winters. Los Angeles has a Mediterranean climate with mild temperatures year round (Peel et al. 2007).

*Case ascertainment*. The outcome was kidney stone presentation, defined as a surgical procedure, hospital admission, and/or at least two emergency room or outpatient clinic visits < 180 days apart for a primary diagnosis of nephrolithiasis using ICD-9 and CPT codes as defined by the Urologic Disease in America Project (Litwin and Saigal 2012). The date of stone presentation was the earliest date of service associated with the nephrolithiasis claim(s) as defined above. Outcomes among unique individuals with more than one presentation for kidney stones during the study period were limited to the earliest occurrence. Individual data were aggregated at the MSA level into daily series of kidney stones counts for the period 2005 to 2011.

*Statistical analysis*. We performed a time-series study using distributed lag nonlinear models (DLNMs) to estimate the relationship between mean daily temperature and kidney stone presentation. Originally developed to evaluate the relationship between temperature and mortality, DLNMs are statistical models that describe associations between exposures and outcomes with potentially nonlinear and delayed effects in time-series data (Armstrong 2006; Gasparrini et al. 2010). We evaluated two aspects of the association between temperature and kidney stone presentation. First, we estimated the relative risk (RR) of kidney stone presentation in association with daily mean temperatures for each day during a 20-day period after the temperature exposure (lag–response). RRs were estimated over the distribution of mean daily temperatures for each MSA relative to a mean daily temperature of 10°C, a moderate temperature that occurred in each of the study locations. Second, we summed the estimated risks for each lag day to estimate the cumulative RR for kidney stone presentation in association with daily mean temperatures during the 20-day period after the temperature exposure (cumulative exposure–response relationship). We used a 20-day lag period based on recent evidence suggesting a short lag time between high temperatures and presentation for kidney stones (Boscolo-Berto et al. 2008; Fletcher et al. 2012).

We built Poisson regression models, allowing for overdispersion for each city as follows:

*Y_t_* ~ Poisson(μ) = α + β*T_t,l_* + *S*(*RH_t_*) + *DOW_t_* + month*_t_* + year*_t_*, [1]

where *t* represents the day of observation; *Y_t_*, the observed stone counts on *t*; α, the intercept; *l*, the lag days; *T_t,l_*, the cross-basis matrix of temperature and lag; *S*(*RH_t_*), the cubic spline of relative humidity on day *t*; and *DOW_t_*, the indicator variable for day of the week at day *t* to control for daily fluctuations in outdoor activities. Month and year are indicator variables to control for season, temperature trends, and differences in the annual at-risk population. We included relative humidity because of its possible independent association with nephrolithiasis as has been reported in previous studies (Boscolo-Berto et al. 2008). For any given temperature, when humidity is low and the air is dry, more water is lost through the skin, thus decreasing urine volume and increasing the supersaturation of calcium and uric acid in the urine.

We used natural cubic splines to smooth the relationships and capture nonlinear associations between temperature and kidney stone diagnoses, and fit the same model for all five study MSAs to avoid overfitting for any particular city. We evaluated one to six knots placed at equal intervals over the range of temperatures and lag days, with the latter natural log-transformed to increase sensitivity for shorter lags. Our final model included the fewest knots needed to capture inflections in the associations and minimize the Akaike information criterion (AIC), specifically two knots for temperature and four for lag (at 2, 3, 4, and 7 days). Locations of temperature knots were Atlanta (6.7°C, 18.9°C), Chicago (–8.9°C, 6.1°C), Dallas (6.5°C, 21.4°C), Los Angeles (13.0°C, 20.6°C), and Philadelphia (3.7°C, 18.4°C). We assessed for differences in MSA mean annual temperature using two-sided analysis of variance tests. Statistical significance was defined as *p* < 0.05. Analyses were performed with R (version 3.0.1; R Project for Statistical Computing; http://www.r-project.org/) using the *dlnm* package (Gasparrini 2011).

*Sensitivity analyses*. We performed several sensitivity analyses to evaluate the robustness of our results given the sensitivity of DLNMs to model choice. First, we used quadratic splines to capture nonlinear effects at temperature extremes. Second, we defined temperature as minimum and maximum daily temperatures to assess whether alternative definitions of temperature exposure changed the estimated associations between temperature and kidney stone presentation. Finally, we increased the lag window to 30 days given the suggestion of longer lag times in previous reports (Boscolo-Berto et al. 2008; Evans and Costabile 2005). The number and location of spline knots for the temperature range and lag period in the models used for the sensitivity analyses with a 20-day lag window were the same as those used for the primary analysis. For models that assessed a 30-day lag, spline knots were placed at 2, 3, 5, and 10 days.

## Results

Between 2005 and 2011, 60,433 patients enrolled in insurance plans that were contained in MarketScan sought medical attention for kidney stones in Atlanta, Chicago, Dallas, Los Angeles, and Philadelphia (Table 1). With the exception of Atlanta and Los Angeles (*p* = 0.09), mean annual temperatures of each city were different (*p* < 0.001).

**Table 1:** Summary statistics for Atlanta, Chicago, Dallas, Los Angeles, and Philadelphia, 2005–2011.

Population characteristic	Atlanta	Chicago	Dallas	Los Angeles	Philadelphia
Kidney stone events (*n*)	12,165	23,699	12,462	7,803	4,304
Mean daily temperature (range)	–5–31	–23–32	–8–36	4–31	–11–33
Days > 30°C (*n*)	16	5	324	6	12
Mean daily relative humidity (range)	54 (13–100)	61 (16–100)	47 (11–100)	42 (5–100)	54 (10–100)
Median age (interquartile range)	53 (43–64)	60 (51–70)	46 (36–57)	42 (32–51)	54 (43–65)
Average annual population enrolled in MarketScan (*n*)	646,037	1,304,449	652,740	685,966	225,771
Average annual kidney stone incidence from 2005 to 2011 per 100,000^*a*^ (range)	274 (229–327)	260 (217–269)	272 (245–339)	167 (146–192)	256 (219–294)
^***a***^The denominator for the average annual incidence of kidney stones is the actual population living in each city enrolled in MarketScan for the entirety of the year.					

**Figure 1:**
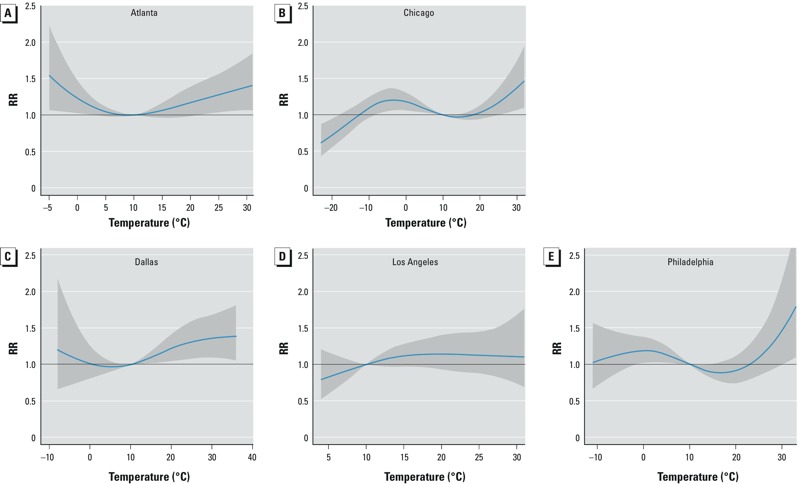
Overall RRs of kidney stone presentation cumulated over a 20-day lag period associated with mean daily temperature (°C) relative to 10°C in Atlanta (*A*), Chicago (*B*), Dallas (*C*), Los Angeles (*D*), and Philadelphia (*E*) from 2005 through 2011. The estimated RRs of kidney stone presentation associated with mean daily temperature cumulated over a 20-day lag period using distributed lag nonlinear models are shown for each city. Two spline knots were placed at equal intervals over the range of temperatures for each city. Locations of temperature knots were as follows: Atlanta (6.7°C, 18.9°C), Chicago (–8.9°C, 6.1°C), Dallas (6.5°C, 21.4°C), Los Angeles (13.0°C, 20.6°C), and Philadelphia (3.7°C, 18.4°C). Four spline knots were placed at equal intervals in the natural log scale of lags (2, 3, 4, and 7 days) to increase sensitivity for shorter lags. The solid blue line is the point estimate at each temperature, and the surrounding gray area the 95% CI.

*Overall cumulative exposure–response relationship*. Associations between mean daily temperature and kidney stone presentation were not monotonic, and there was variation in the shape of the exposure–response curves and the strength of associations at different temperatures. However, in most cases, RRs increased for temperatures above the reference value of 10°C (Figure 1). RRs for a kidney stone presentation cumulated over a 20-day period associated with a mean daily temperature of 30°C compared with 10°C were 1.38 in Atlanta [95% confidence interval (CI): 1.07, 1.79], 1.37 in Chicago (95% CI: 1.07, 1.76), 1.36 in Dallas (95% CI: 1.10, 1.69), 1.11 in Los Angeles (95% CI: 0.73, 1.68), and 1.47 in Philadelphia (95% CI: 1.00, 2.17). The temperatures > 10°C at which statistically significant associations were first observed varied among the cities (Table 2). Heterogeneity was also noted at the limits of the temperature ranges. In Dallas, the excess RR of kidney stone presentation stabilized at 36–39% as temperatures increased > 30°C. For Atlanta, Chicago, and Philadelphia, the risk of kidney stone presentation increased throughout the upper temperature range of each city. Kidney stone presentations also were positively associated with temperatures < 2°C in Atlanta, and < 10°C in Chicago and Philadelphia. Relative humidity was not a statistically significant predictor of the risk of kidney stone presentation (data not shown).

**Table 2:** RR (95% CI) of kidney stone presentation cumulated over a 20-day lag period associated with mean daily temperature (°C) relative to 10°C based on data from privately insured residents of Atlanta, Chicago, Dallas, Los Angeles, and Philadelphia, 2005–2011.

Mean daily temperature (°C)	Atlanta	Chicago	Dallas	Los Angeles	Philadelphia
36	NA	NA	1.39 (1.06, 1.82)*	NA	NA
34	NA	NA	1.38 (1.08, 1.77)*	NA	NA
32	NA	1.46 (1.10, 1.95)*	1.37 (1.09, 1.72)*	NA	1.68 (1.07, 2.64)*
30	1.38 (1.07, 1.79)*	1.37 (1.07, 1.76)*	1.36 (1.10, 1.69)*	1.11 (0.73, 1.68)	1.47 (1.00, 2.17)*
28	1.34 (1.06, 1.69)*	1.28 (1.04, 1.58)*	1.35 (1.10, 1.65)*	1.12 (0.81, 1.54)	1.30 (0.93, 1.80)
26	1.30 (1.05, 1.60)*	1.20 (1.01, 1.44)*	1.32 (1.09, 1.62)*	1.12 (0.86, 1.47)	1.16 (0.87, 1.54)
24	1.26 (1.03, 1.53)*	1.14 (0.99, 1.31)	1.30 (1.07, 1.57)*	1.13 (0.89, 1.44)	1.05 (0.82, 1.34)
22	1.21 (1.00, 1.46)*	1.08 (0.96, 1.21)	1.26 (1.06, 1.50)*	1.14 (0.90, 1.43)	0.97 (0.77, 1.21)
20	1.17 (0.98, 1.39)	1.03 (0.95, 1.13)	1.22 (1.04, 1.42)*	1.14 (0.93, 1.40)	0.91 (0.75, 1.12)
18	1.12 (0.97, 1.31)	1.00 (0.94, 1.07)	1.17 (1.03, 1.33)*	1.14 (0.96, 1.36)	0.89 (0.74, 1.06)
16	1.08 (0.96, 1.22)	0.98 (0.93, 1.03)	1.12 (1.02, 1.23)*	1.13 (0.97, 1.31)	0.89 (0.77, 1.03)
14	1.04 (0.97, 1.13)	0.97 (0.94, 1.01)	1.07 (1.01, 1.14)*	1.10 (0.97, 1.25)	0.91 (0.83, 1.01)
12	1.02 (0.98, 1.05)	0.98 (0.96, 1.00)*	1.03 (1.00, 1.06)*	1.06 (0.97, 1.15)	0.95 (0.90, 1.00)*
10	Referent	Referent	Referent	Referent	Referent
8	1.00 (0.97, 1.03)	1.03 (1.01, 1.05)*	0.98 (0.95, 1.00)	0.93 (0.83, 1.05)	1.06 (1.01, 1.11)*
6	1.02 (0.97, 1.08)	1.07 (1.02, 1.11)*	0.97 (0.92, 1.02)	0.86 (0.66, 1.12)	1.11 (1.02, 1.21)*
4	1.07 (0.99, 1.16)	1.11 (1.04, 1.18)*	0.97 (0.88, 1.07)	0.79 (0.52, 1.21)	1.15 (1.03, 1.30)*
2	1.14 (1.01, 1.29)*	1.15 (1.05, 1.25)*	0.99 (0.85, 1.15)	NA	1.18 (1.03, 1.35)*
0	1.23 (1.03, 1.47)*	1.18 (1.06, 1.31)*	1.01 (0.81, 1.27)	NA	1.19 (1.02, 1.38)*
–2	1.34 (1.04, 1.73)*	1.20 (1.07, 1.35)*	1.05 (0.77, 1.43)	NA	1.18 (0.99, 1.40)
–4	1.47 (1.06, 2.05)*	1.21 (1.06, 1.37)*	1.09 (0.73, 1.63)	NA	1.16 (0.94, 1.42)
–6	NA	1.19 (1.04, 1.35)*	1.15 (0.70, 1.89)	NA	1.12 (0.87, 1.45)
–8	NA	1.15 (1.01, 1.31)*	1.20 (0.66, 2.20)	NA	1.09 (0.79, 1.49)
–10	NA	1.10 (0.96, 1.25)	NA	NA	1.05 (0.71, 1.55)
–12	NA	1.03 (0.90, 1.18)	NA	NA	NA
–14	NA	0.95 (0.81, 1.12)	NA	NA	NA
–16	NA	0.87 (0.72, 1.06)	NA	NA	NA
–18	NA	0.80 (0.63, 1.00)	NA	NA	NA
–20	NA	0.72 (0.55, 0.95)*	NA	NA	NA
–22	NA	0.65 (0.47, 0.90)*	NA	NA	NA
Estimates of RR and CIs were not available (NA) for temperatures that were outside of the temperature range for each city. Distributed lag nonlinear models allowing for overdispersion were used to estimate the results for each city: *Y*_*t*_ ~ Poisson(μ) = α + β*T*_*t,l*_ + *S*(*RH*_*t*_) + *DOW*_*t*_ + month_*t*_ + year_*t*_, where *t* = day of observation; *Y*_*t*_ = observed stone counts on *t*; α = intercept; *l* = lag days; *T*_*t,l*_ = cross-basis matrix of temperature and lag; *S*(*RH*) = cubic spline of relative humidity; *DOW*_*t*_ = indicator variable for day of the week at *t* to control for daily fluctuations in outdoor activities; month and year are indicator variables to control for season, temperature trends, and differences in the annual at-risk population. *p < 0.05.					

**Figure 2:**
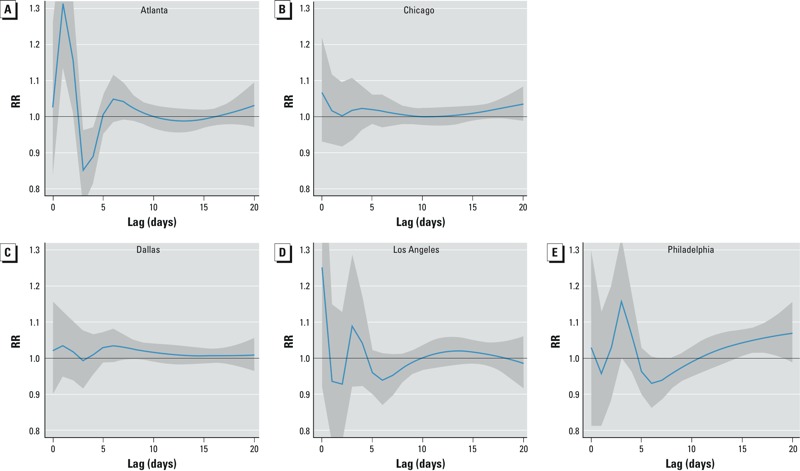
Lag response between a 30°C (mean) day and kidney stone presentation relative to 10°C over a 20-day period in Atlanta (*A*), Chicago (*B*), Dallas (*C*), Los Angeles (*D*), and Philadelphia (*E*) from 2005 through 2011. For each city, the estimated RRs of kidney stone presentation in association with a daily mean temperature of 30°C (relative to 10°C) for each lag day from the temperature exposure during a 20-day period are shown. We used distributed lag nonlinear models to estimate the RRs and placed two spline knots at equal intervals over the range of temperatures for each city. Locations of temperature knots were as follows: Atlanta (6.7°C, 18.9°C), Chicago (–8.9°C, 6.1°C), Dallas (6.5°C, 21.4°C), Los Angeles (13.0°C, 20.6°C), and Philadelphia (3.7°C, 18.4°C). We placed four spline knots at equal intervals in the natural log scale of lags (2, 3, 4, and 7 days) to increase sensitivity for shorter lags. The solid blue line is the RR at each lag day from the exposure, and the surrounding gray area the 95% CI.

*Lag response*. We estimated bimodal increases in the RR of kidney stone presentation for days in which the temperature was 30°C relative to days with mean temperatures of 10°C. The strongest association between kidney stone presentation and a daily mean temperature of 30°C versus 10°C was estimated for lags ≤ 3 days and a second peak was estimated at 4 to 6 days (Figure 2). Periods of increased risk were followed immediately by days of lower risk. The RRs of kidney stone presentation after hot days at 10–20 days lag were heterogeneous. A trend of increased risk was found in Philadelphia from 10–20 days, and in Atlanta and Chicago from 15–20 days, whereas the risk in Dallas and Los Angeles varied around the null after 10 days.

**Figure 3:**
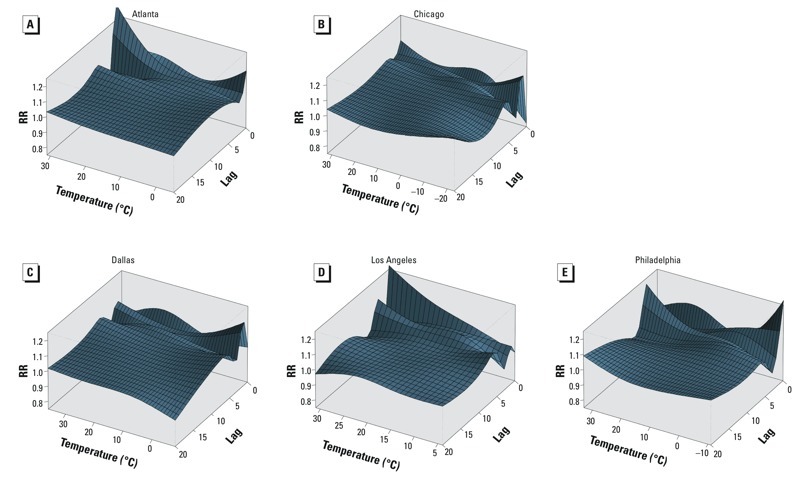
Risk of kidney stone presentation relative to 10°C along temperature and a 20-day lag period in Atlanta (*A*), Chicago (*B*), Dallas (*C*), Los Angeles (*D*), and Philadelphia (*E*) from 2005 through 2011. The three-dimensional relationships include temperature (*x*-axis), lag (*z*-axis), and RR of kidney stone presentation (*y*-axis). The point estimate of the RR of kidney stone presentation at each point along the temperature range and lag window is shown using 10°C as the reference temperature. We used distributed lag nonlinear models to estimate the RRs and placed two spline knots at equal intervals over the range of temperatures for each city. Locations of temperature knots were as follows: Atlanta (6.7°C, 18.9°C), Chicago (–8.9°C, 6.1°C), Dallas (6.5°C, 21.4°C), Los Angeles (13.0°C, 20.6°C), and Philadelphia (3.7°C, 18.4°C). We placed four spline knots at equal intervals in the natural log scale of lags (2, 3, 4, and 7 days) to increase sensitivity for shorter lags.

*RR along exposure–response curve and lag*. We constructed three-dimensional graphs to demonstrate simultaneously the relationships along temperature and lag (Figure 3). Consistent trends of increasing RR of kidney stone presentation were observed within 7 days of high temperatures across cities. However, CIs cannot be represented in these figures, and therefore the precision of the estimates cannot be appreciated.

*Sensitivity analyses*. The shapes of the overall cumulative exposure–response and lag response relationships between temperature and nephrolithiasis using quadratic splines were similar to the natural cubic splines and did not reveal any nonlinear relationships at temperature range limits. This suggests that these relationships were adequately captured by natural cubic splines, which were chosen for the main model because of their more conservative smoothing of data and the gain of a degree of freedom. The patterns of the overall cumulative exposure–response relationship and the distribution of risk across time in the lag response estimations were consistent whether mean, maximum, or minimum daily temperatures were used. However, compared with mean daily temperature, the precision of the estimates for maximum temperatures were lower and the AIC values were larger for minimum temperatures (data not shown). Increasing the lag window to 30 days also decreased the precision of the temperature–nephrolithiasis association as evidenced by the wider CIs in the overall cumulative exposure–response curves (see Supplemental Material, Figure S1). With a lag window of 30 days, a slight decrease in the RR of kidney stone presentation was observed at lags of 20–25 days from a 30°C day in Dallas, Los Angeles, and Philadelphia (see Supplemental Material, Figure S2); however, the RR of nephrolithiasis over this lag subperiod was < 3% (95% CI: 1.00, 1.05).

## Discussion

We observed that as daily temperatures increased to > 10°C, the risk of kidney stone presentation over the next 20 days also increased in most cities. The lag between high daily temperatures and the risk of kidney stone presentation was short, with the maximum risk occurring ≤ 3 days of temperature exposure. Our estimations suggest that there is a graded increase in the risk of patients seeking medical care for kidney stones as average daily temperatures increase and that the time between hot days and kidney stone presentation is short.

We hypothesize that the estimated RRs of kidney stone presentation across a range of temperatures might represent patients presenting with calcium-based kidney stones, which are the most common type of stones in the United States and other developed countries. Patients who are at risk for developing calcium stones are those with calcium apatite deposits in the basement membranes of the thin loops of Henle (Randall’s plaques) or in the collecting ducts of the nephron. The rate at which calcium apatite deposits form is currently unknown, but it is likely these deposits build up over months to years, and once they ultimately erode through the urothelium, serve as the nidus for stone formation within the urinary space (Evan et al. 2003, 2007). Dehydration, the proposed causal mechanism through which high temperatures would act, increases the supersaturation of calcium and uric acid, thus promoting calcium stone formation on the apatite deposits. Modeling and laboratory experiments suggest that stones grow over a span of hours in the proper urinary environment and that growth rates are dramatically increased by increasing urinary supersaturation of calcium (Borissova et al. 2010; Costa-Bauza et al. 2005). An alternative hypothesis is that temperature extremes may cause patients with a diverse group of conditions to seek medical care, and the risks we estimated are not unique to kidney stones.

*Overall cumulative exposure–response relationship*. The association between higher daily temperatures and an increased risk of kidney stone presentation was generally consistent among different cities. The RR of nephrolithiasis began to rise significantly at warm temperatures (e.g., 24–26°C) in Atlanta, Chicago, and Dallas, and continued to increase with rising temperatures. At 30°C in Atlanta, Chicago, and Dallas, the three cities with the highest number of kidney stone events and hence the most precise estimates), the RR of a kidney stone presentation varied by only 3%, and was 36–39% higher than at 10°C. There were, however, some differences among the cities. The risk of stone presentation was 47% higher at 30°C than at 10°C in Philadelphia, which had the fewest number of patients and thus less precise estimates. In Los Angeles, the RR of kidney stone presentation increased between temperatures of 10°C and 15°C, but did not reach statistical significance and did not rise further after 15°C.

Our estimates suggest a possible ceiling effect in Dallas, where a 36–39% excess risk of nephrolithiasis was estimated as temperatures rose to > 30°C. One explanation for the ceiling effect is that the population adapts to the local climate: At high temperatures in developed cities, people spend more time indoors and increase their fluid intake, thus mitigating the effect of ambient temperature extremes. It is also possible the association flattened off because it was constrained to do so by the model. We caution against overinterpreting the associations we estimated for temperature extremes in part because we used cubic splines in our models to smooth data that are constrained to be linear at the extremes of temperatures where data are sparse.

An increased risk of kidney stone presentation was estimated for cold temperatures in Atlanta, Chicago, and Philadelphia. To our knowledge, an association between very cold temperatures and kidney stones has been reported in only one previous study. In that study conducted in Norway, which has cold winters and mild summers, Laerum (1983) reported that renal colic occurred more frequently in winter months than in summer months. In a manner similar to that of high heat, people likely adapt to very cold temperatures by spending more time inside and thus have a risk of nephrolithiasis associated with higher indoor temperatures. An ancillary explanation is that cold weather is associated with behaviors that increase the risk of nephrolithiasis, such as the consumption of stone-promoting foods and beverages or decreased fluid intake.

In the only other study that examined the exposure–response relationship between daily temperatures and nephrolithiasis of which we are aware, Fletcher et al. (2012) estimated a 6% increase in the odds of hospital admission for nephrolithiasis for each 2.8°C increase in daily summer temperatures. This equates to a 42% higher odds of kidney stone presentation at 30°C relative to 10°C, which is similar to the RRs we estimated in our analysis, which included different geographic areas and spanned all seasons. Although the present study was performed in the United States, the cities included in the analysis have climates that are representative of those found throughout the world. Given the increase of nephrolithiasis globally (Romero et al. 2010) and the increase in global temperatures (Marcott et al. 2013), more studies are needed to estimate the association between temperature and kidney stones in other countries. We hypothesize that the association between daily temperature and kidney stone presentations is similar in different regions of the world, as it is with temperature and mortality (Armstrong 2006; Gasparrini et al. 2012; Guo et al. 2011).

*Lag response*. The lag between high temperatures and kidney stone presentation was short, with the maximum risk occurring ≤ 3 days of exposure and persistence of a mildly elevated RR at 10–20 days in Philadelphia. The lag response we observed is strikingly similar to Fletcher et al.’s (2012) case-crossover study in which the maximum odds for admission for kidney stones after high daily temperatures also was observed at 3 days. Although lag response was not directly measured, Boscolo-Berto et al. (2008) estimated the correlation between the average temperatures of the 15, 30, 45, and 60 days preceding presentation of patients with renal colic in northern Italy. Renal colic was most strongly correlated with temperatures over the preceding 15 days. Finally, Evans and Costabile (2005) noted that the mean time to symptomatic stone occurrence, defined by the period of time between arrival of soldiers in the Kuwait desert and presentation with a symptomatic stone, was 93 days. Although, as concluded by the authors, these results appear to support a longer time interval between exposure to hot temperatures and symptomatic kidney stone presentation, closer analysis suggests the lag response is consistent with our observations. Troops were deployed in March when average daily temperatures in Kuwait were 20°C. Few symptomatic stones were observed until the end of May, when daily temperatures approached 35°C, and the highest incidence of stones was noted in June through August, when average daily temperatures were 36–38°C. Although the RR of kidney stone presentation associated with daily temperatures we estimated do not prove causation, they are consistent with the few reports on the short lag between temperature exposure and kidney stone presentation (Boscolo-Berto et al. 2008; Evans and Costabile 2005; Fletcher et al. 2012) and raise questions about the rate at which kidney stones might develop *in vivo*.

Within 7–10 days of the temperature exposure, we observed that increases in the RR of kidney stone presentation were immediately followed by decreases in RR. We attribute this phenomenon, which has been observed in the association between temperature and mortality (Armstrong 2006; Guo et al. 2011), to the “harvesting” of nephrolithiasis cases in subjects who would have formed stones at some future time, but for whom heat caused the event to occur earlier. We hypothesize that harvesting of susceptible cases reflects the causal mechanism of stone formation and explains the short lag between hot days and stone presentation. In patients who are predisposed to stone formation (e.g., those with Randall’s plaques), low urinary volume resulting from sweating on hot days may result in spontaneous stone nucleation and/or cause small asymptomatic stones to grow large enough to become symptomatic.

*Limitations*. We acknowledge the present study’s limitations. First, we do not know if the association between high daily temperatures and kidney stone presentation reflects the time of stone formation or the detachment of previously formed stones from the urothelium. In addition, because this study included only subjects who formed stones, the association we observed between temperature and nephrolithiasis only applies to those who are at risk for stone formation, such as those with Randall’s plaques or calcium apatite deposits in the collecting duct of the nephron.

Second, misclassification of exposure is possible. Temperatures may vary within an MSA, and we do not know which subjects had access to resources that lessen the effect of heat and cold. Access to air conditioning should bias our estimates of association at high temperatures toward the null. In addition, commercially insured patients are less likely to work in jobs that require exposure to ambient temperatures (Park 2000), which should also bias our estimates toward a null association.

An advantage of time-series designs is that factors that are constant in the short time frame of the exposure–response relationship (e.g., race, sex, age) should not confound the association between temperature and the measured outcome (Gasparrini and Armstrong 2010). However, the effect of temperature on nephrolithiasis may differ in certain patient subgroups. For example, the young and the elderly may be more sensitive to heat, and the effect of heat on urine chemistries and volume may differ by sex (Parks et al. 2003). However, stratification or restricting the analyses to particular patient characteristics was not reasonable in the present study because of the reduction in power that would result from the much smaller sample size. Further studies to determine whether, race, age, or sex modifies the effect of temperature will help elucidate particular groups of patients who are especially vulnerable to heat-mediated nephrolithiasis.

*Implications*. Global temperatures between 2000 and 2009 were warmer than 82% of temperatures over the last 11,300 years (Marcott et al. 2013). Continued greenhouse gas emissions are projected to further increase global average temperatures by 1–4.5°C during the 21st century (Meinshausen et al. 2011; Solomon et al. 2007). Brikowski et al. (2008) predicted how the geographic distribution and prevalence of nephrolithiasis would change in the United States due to temperature increases caused by greenhouse gas emissions. Using theoretical models, Brikowski et al. (2008) estimated that 1.6–2.2 million new cases of nephrolithiasis will result from increases in mean annual temperatures by 2050 and that > 70% of the U.S. population would live in “high-risk” zones for nephrolithiasis by 2095. However, their projections relied on speculative models of temperature dependence of stone risk and used mean annual temperature to define temperature exposure. The present study provides an empiric model of the temperature dependence of kidney stone presentation in diverse geographic areas with different climates.

Our observations of the association between hot days and kidney stone presentation also call into question whether mean annual temperature is the most appropriate metric of temperature exposure for predicting the future distribution and prevalence of nephrolithiasis under different climate change scenarios. Mean annual temperature has been used universally to explain geographic differences in nephrolithiasis prevalence (Chen et al. 2000; Soucie et al. 1996). However, we believe mean annual temperature is an oversimplification of temperature exposure because it does not accurately reflect the proportion of hot days experienced by a population. Our observations of the prevalence of nephrolithiasis and mean annual temperature in Atlanta and Los Angeles illustrate this concept. The prevalence of nephrolithiasis in Atlanta was almost two times greater than in Los Angeles, but the mean annual temperature of both Atlanta and Los Angeles was 17°C. However, Atlanta, on average, had 53 days each year with mean 24-hr temperatures > 27°C, compared with 10 days for Los Angeles. Thus, mean annual temperature may inadequately reflect the heat experienced by a population. Given the association between high daily temperatures and kidney stone presentation we observed, we propose that the number of hot days (e.g., > 30°C in temperature) in a given year is a better predictor of the prevalence of nephrolithiasis than mean annual temperature. Future studies are needed to validate our findings in the general population and to determine the best definition of high ambient temperature exposure as it relates to the prevalence of nephrolithiasis.

## Conclusions

In general, kidney stone presentations increased with higher daily mean temperatures, with the strongest associations estimated for lags of only a few days. These findings further support the hypothesis of an adverse effect of high temperatures on nephrolithiasis. Given the threat of climate change and the increasing prevalence of nephrolithiasis globally, more studies are needed to determine how daily temperatures affect the risk of kidney stone presentation in different populations and what genetic, behavioral, and environmental factors modify the effect.

## Supplemental Material

(249 KB) PDF
