# Supplementary material for: Daily Mean Temperature and Clinical Kidney Stone Presentation in Five U.S. Metropolitan Areas: A Time-Series Analysis
Source: Environ Health Perspect. 2014 Jul 10;122(10):1081–7. doi: 10.1289/ehp.1307703 (PMC4181925; doi:10.1289/ehp.1307703)

Environmental Health Perspectives doi:10.1289/ehp.1307703

Supplemental Material

Daily Mean Temperature and Clinical Kidney Stone Presentation in Five U.S. Metropolitan Areas: A Time-Series Analysis

Gregory E. Tasian, Jose E. Pulido, Antonio Gasparrini, Christopher S. Saigal, Benjamin P. Horton, J. Richard Landis, Rodger Madison, and Ron Keren for the Urologic Diseases in America Project

Figure S1. Overall relative risk of kidney stone presentation cumulated over 30 day lag period associated with mean daily temperature (°C) relative to 10°C in Atlanta (A), Chicago (B), Dallas (C), Los Angeles (D), and Philadelphia (E) from 2005-2011. The estimated relative risk of kidney stone presentation associated with mean daily temperature cumulated over a 30 day lag period using distributed lag non-linear models are shown. Two spline knots were placed at equal intervals over the range of temperatures for each city. Locations of temperature knots were: Atlanta (6.7°C, 18.9°C), Chicago (-8.9°C, 6.1°C), Dallas (6.5°C, 21.4°C), Los Angeles (13.0°C, 20.6°C), Philadelphia (3.7°C, 18.4°C). Four spline knots were placed at equal intervals in the natural log scale of lags (2, 3, 5, and 10 days) to increase sensitivity for shorter lags. The solid line is the point estimate at each temperature and the surrounding grey area represents the 95% CI.

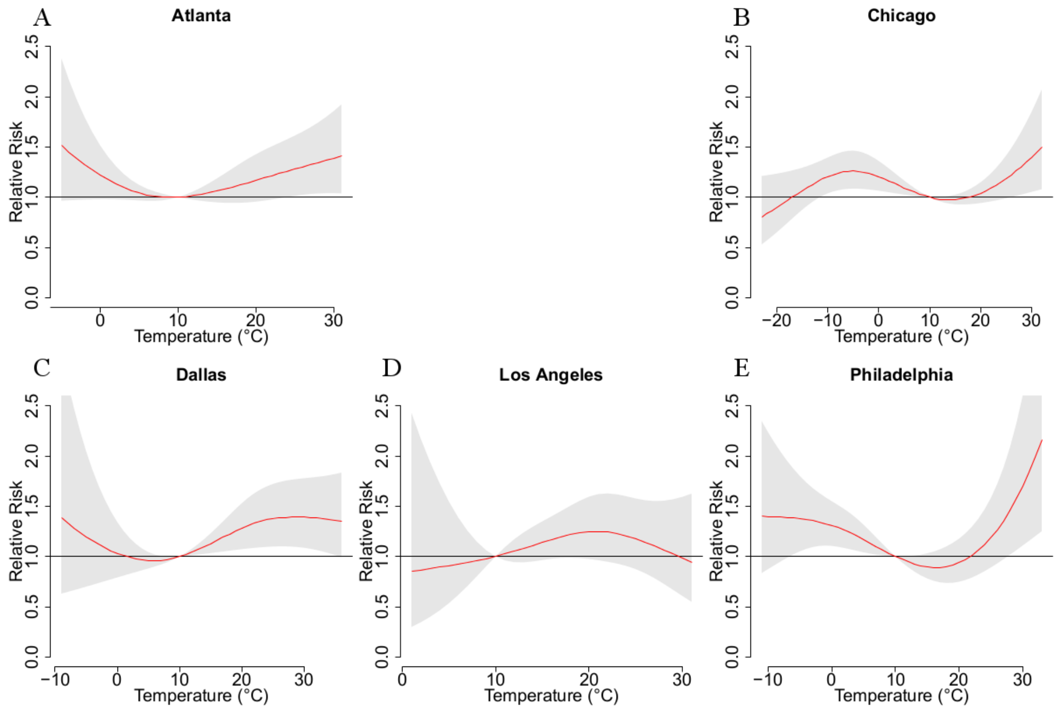

Figure S2. Lag-response between a 30°C (mean) day and kidney stone presentation relative to 10°C over a 30 day period in Atlanta (A), Chicago (B), Dallas (C), Los Angeles (D), and Philadelphia (E) from 2005-2011. For each city, the estimated relative risks of kidney stone presentation in association with a daily mean temperature of 30°C (relative to 10°C) for each lag day from the temperature exposure during a 30-day period are shown. We used distributed lag non-linear models to estimate the relative risks and placed two spline knots at equal intervals over the range of temperatures for each city. Locations of temperature knots were: Atlanta (6.7°C, 18.9°C), Chicago (-8.9°C, 6.1°C), Dallas (6.5°C, 21.4°C), Los Angeles (13.0°C, 20.6°C), Philadelphia (3.7°C, 18.4°C). We placed four spline knots at equal intervals in the natural log scale of lags (2, 3, 5, and 10 days) to increase sensitivity for shorter lags. The solid line is the relative risk at each lag day from the exposure and the surrounding grey area represents the 95% CI.

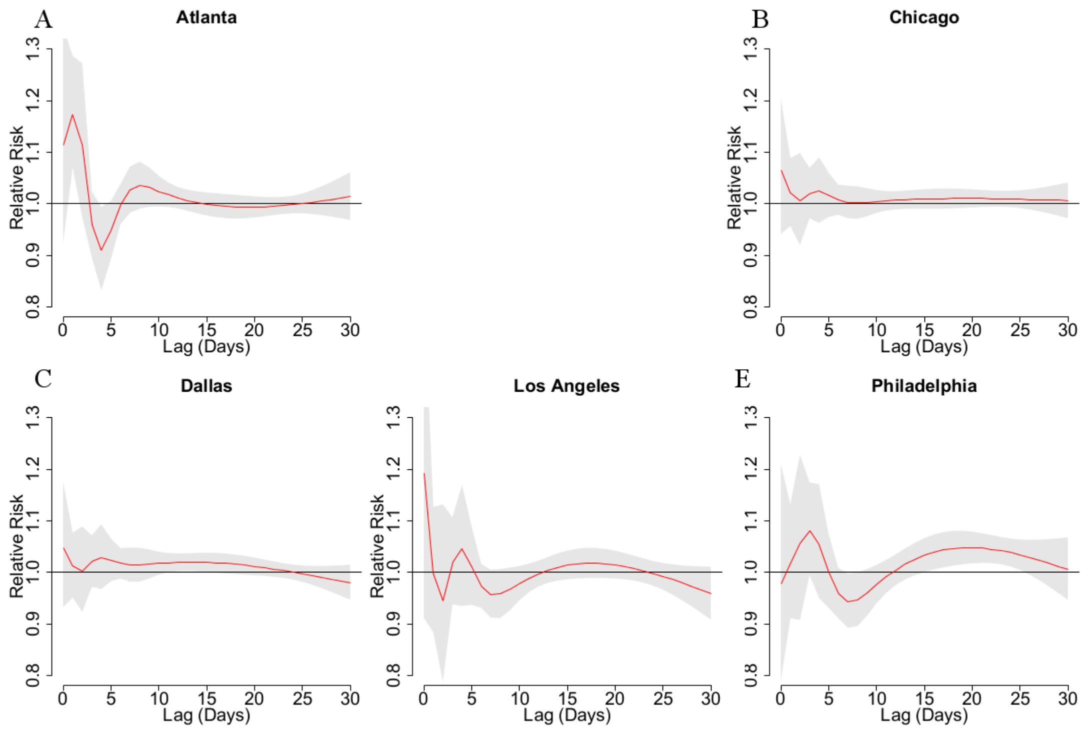

Supplement: (249 KB) PDF [file ehp.1307703.s001.508.pdf]
